# Aerobic scope explains individual variation in feeding capacity

**DOI:** 10.1098/rsbl.2015.0793

**Published:** 2015-11

**Authors:** Sonya K. Auer, Karine Salin, Graeme J. Anderson, Neil B. Metcalfe

**Affiliations:** Institute of Biodiversity, Animal Health and Comparative Medicine, University of Glasgow, Graham Kerr Building, Glasgow G12 8QQ, UK

**Keywords:** energy metabolism, fitness, food intake, maximum metabolic rate, *Salmo trutta*, standard metabolic rate

## Abstract

Links between metabolism and components of fitness such as growth, reproduction and survival can depend on food availability. A high standard metabolic rate (SMR; baseline energy expenditure) or aerobic scope (AS; the difference between an individual's maximum and SMR) is often beneficial when food is abundant or easily accessible but can be less important or even disadvantageous when food levels decline. While the mechanisms underlying these context-dependent associations are not well understood, they suggest that individuals with a higher SMR or AS are better able to take advantage of high food abundance. Here we show that juvenile brown trout (*Salmo trutta*) with a higher AS were able to consume more food per day relative to individuals with a lower AS. These results help explain why a high aerobic capacity can improve performance measures such as growth rate at high but not low levels of food availability.

## 1. Introduction

Metabolic rate reflects the energetic cost of fuelling all processes and functions needed to support life [1]. At the very minimum, an organism must expend energy on basic functions that maintain homeostasis. Above this baseline expenditure, or standard metabolic rate (SMR), organisms must grow, reproduce and evade predators but within the bounds set by the upper limits of their maximum metabolic rate (MMR); the difference between MMR and SMR is referred to as aerobic scope (AS). Metabolic rates are therefore key physiological traits underlying the performance of organisms [2].

Consistent individual differences in metabolism are associated with components of fitness such as growth, reproduction and survival [3]. However, these relationships can vary depending on environmental conditions, most notably with food availability, whereby a high SMR or AS is often beneficial when food is abundant or easily accessible but less important or even disadvantageous when food availability declines [3,4]. While the mechanisms underlying these context-dependent associations are not well understood, they suggest that individuals with higher SMR or AS are better able to take advantage of high food abundance. At the whole organism level, an individual's SMR and AS have been shown to influence a number of performance measures such as its locomotor ability [5], boldness, competitive dominance and territorial aggression [2] that may influence food acquisition to varying extents. However, we know very little about the importance of these metabolic traits for non-behavioural aspects of foraging such as feeding capacity that directly influence energy intake.

There is some evidence suggesting that individuals with a higher SMR can digest meals faster [6], which implies they may be able to consume more food per day. The metabolic costs of ingestion, digestion, absorption and assimilation (specific dynamic action or SDA) increase with meal size [7], so an individual's AS may also limit its food intake at higher food levels, but this prediction remains untested. We examined whether variation in maximum food consumption rates among juvenile brown trout (*Salmo trutta*) is explained by differences in their SMR and/or AS. Trout with a higher SMR and AS are known to have higher growth rates under *ad libitum* but not lower food levels [4], so differences in feeding capacity associated with these metabolic traits may explain why the effects of metabolism depend on food levels.

## 2. Material and methods

Metabolic traits and feeding capacity were measured at 10°C in juvenile brown trout (see the electronic supplementary material for fish care and feeding regime). SMR was measured over a 24 h period as the rate of oxygen consumption using continuous flow-through respirometry (electronic supplementary material; [4]). MMR was then estimated using an exhaustive chase protocol followed immediately by measurement of excess post-exercise oxygen consumption using intermittent flow-through respirometry (electronic supplementary material; [4]). The AS for each fish was calculated as the difference between its MMR and SMR. Fish were then weighed while under a mild anaesthetic (40 mg l^−1^ benzocaine), returned to their normal feeding regime (electronic supplementary material), and given one week to recover from their metabolic rate measurements before the feeding trials began.

The feeding capacity of each fish was determined over a 3-day trial in which they were fed *ad libitum* twice daily, in early morning and late afternoon to simulate the peak drift frequencies they experience in the wild [4]. Trials lasted for 3 days to allow for multiple measures of each individual without the risk that estimates could be influenced by changes in their body mass and metabolic rates [8]. Fish were not fed for 48 h prior to the trial to ensure that their guts were evacuated, and they were weighed, as described above, the day before the trial began. After feeding, fish were left undisturbed and given 1 h to consume their meal before it was siphoned from their tank [4,9]. Meal size was calculated as the difference between the number of pellets fed to each fish and the number remaining after 1 h. Trout can only consume two meals per day at 10°C [9], so the two meals were summed for each day, converted to mg (3 mg/pellet), and then averaged over the 3-day trial to calculate average daily maximum food consumption.

Linear mixed models were used to relate each metabolic trait—SMR, MMR and AS—to body mass, and then to examine whether these metabolic traits explained individual variation in average daily food consumption. All metabolic traits were a positive function of body mass but showed almost twofold variation after controlling for mass (electronic supplementary material, table S1 and figure S1), so residuals derived from the mixed models for each trait were used as predictors in the following analyses of feeding capacity. Since SMR was somewhat correlated with the other two traits (SMR versus MMR: Pearson's *r*_28_ = −0.33, *p* = 0.08; SMR versus AS: *r*_28_ = −0.55, *p* = 0.001), we first tested the predictive ability of each of the three metabolic traits separately and then together (first SMR and MMR, then SMR and AS) in the same models (variation inflation factor <1.5). Interactions between metabolic traits were included, but were subsequently removed since non-significant. Results for models including AS were the same for those including MMR (electronic supplementary material) as the two metabolic rate measures were highly correlated (*r*_28_ = 0.96, *p* < 0.001), so only results for models including AS are reported in the main text. The 30 fish were processed in two batches of 15, so all models included batch number as a random effect. Analyses were conducted using SPSS v. 22 (SPSS Inc., Chicago, IL, USA).

## 3. Results

**Figure 1.**
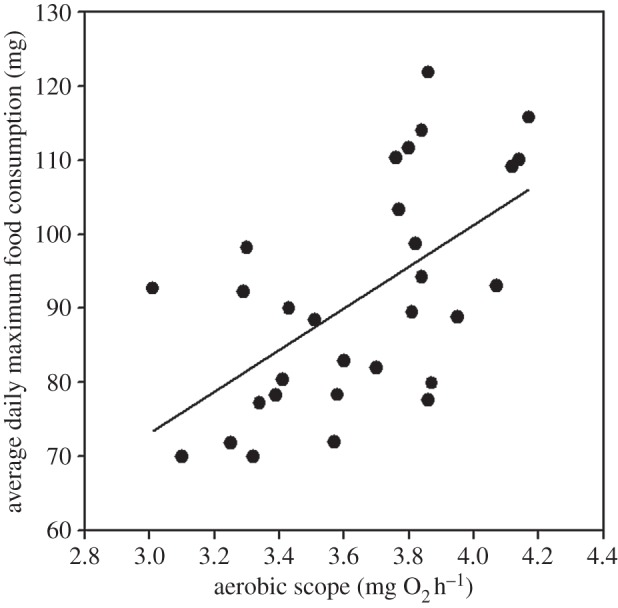
Average daily maximum food consumption as a function of AS in juvenile brown trout (*Salmo trutta*) fed *ad libitum* (*R*^2^ = 0.35). Plotted are partial residuals evaluated at the mean body mass (6.5 g) and SMR (0.66 mg O_2_ h^−1^).

Fish were able to consume an average of 1.40 ± 0.04% (mean ± 1 s.e.) of their body mass per day, but showed significant variation (range 1.02–1.78%). Larger fish were able to consume more food, but after controlling for size, individual differences were explained by their metabolic traits but to varying degrees: average daily consumption (mg) was greater in individuals with a higher AS (*F*_1,26.0_ = 8.77, *p* = 0.006; body mass: *F*_1,26.0_ = 127.5, *p* < 0.001) but was not a function of SMR (*F*_1,26.1_ = 0.53, *p* = 0.47; body mass: *F*_1,26.1_ = 97.2, *p* ≤ 0.001) when the effect of each metabolic trait was considered separately. Results were the same when considering both SMR and AS together in the same model: average daily food consumption was higher in individuals with a higher AS (figure 1; *F*_1,25.0_ = 9.1, *p* = 0.006) after controlling for the effects of body mass (*F*_1,25.1_ = 127.5, *p* < 0.001); however, mass-independent SMR explained none of the remaining variation (*F*_1,25.0_ = 1.0, *p* = 0.33).

## 4. Discussion

Our key finding was that feeding capacity was a positive function of AS; individual variation in AS was associated with twofold variation in how much food fish of a given size were able to consume per day. AS represents the overall capacity for oxygen supply to the tissues for subsequent ATP production but is limited by the gas transport abilities of both the respiratory and cardiovascular systems [10]. Organisms cannot simultaneously meet all the potential aerobic demands of their many organ systems [11]. Thus, the metabolic cost of SDA, while consuming variable proportions of the aerobic budget among species, often comes at the expense of other functions such as locomotion [11,12]. Given that the costs of SDA increase with meal size [7], our results, while correlational, suggest that AS constrains the SDA response and therefore food consumption rates. Growth is highly dependent on food intake, so this constraint may explain why individuals with a higher AS can grow faster under *ad libitum* conditions, as previously demonstrated in our study species [4]. We might expect these constraints to be particularly acute in species with a relatively low AS [13], or in juveniles that need to grow fast but, because of physiological scaling limitations, must allocate a disproportionate amount of energy towards their SMR at the expense of their AS [14]. Nevertheless, food intake has obvious ramifications for the performance of all organisms, so differences in AS among individuals likely have important consequences for their fitness [15].

SMR explained none of the individual variation in feeding capacity. This is surprising because SMR is thought to reflect the idling costs of the metabolic machinery needed to drive important functions such as growth and digestion [2], and is known to change in response to food levels [8]. Our results also appear in contrast to those of Millidine *et al*. [6], who found that individual variation in SMR was associated with faster digestion, so should permit greater food consumption rates. However, they measured the SDA response to a small ration size (0.30% when compared with 1.4% body mass here), so together these results suggest that SMR is important to feeding capacity at lower but not higher food levels.

Performance measures such as locomotor ability [5], competitive dominance [16] and feeding capacity (this study) are a positive function of AS. Given these advantages, we might expect selection to favour a higher AS, yet see up to twofold variation among individuals [4]. The persistence of such variation suggests that there might be costs to a higher AS, such as higher production of harmful reactive oxygen species [17]. Additionally, while increased food consumption rates facilitated by a higher AS can lead to a higher growth rate, this may trade off against growth efficiency [18]. Individual differences in AS may therefore represent alternative metabolic strategies for coping with fluctuations in environmental factors such as food availability, but this waits further testing.

## Supplementary Material

Electronic supplementary material

## Data accessibility

Data are available in the electronic supplementary material.
