# Supplementary material for: Aerobic scope explains individual variation in feeding capacity
Source: Biol Lett. 2015 Nov;11(11):20150793. doi: 10.1098/rsbl.2015.0793 (PMC4685545; doi:10.1098/rsbl.2015.0793)
Supplement: Electronic supplementary material [file rsbl20150793supp1.docx]

***Electronic supplementary material***

**Title:** Aerobic scope explains individual variation in feeding capacity

**Authors and affiliations:** Sonya K. Auer^1^, Karine Salin, Graeme J. Anderson and Neil B. Metcalfe

Institute of Biodiversity, Animal Health and Comparative Medicine, Graham Kerr Building, University of Glasgow, Glasgow G12 8QQ United Kingdom

^1^ Manuscript correspondence: Sonya K. Auer, Institute of Biodiversity, Animal Health and Comparative Medicine, University of Glasgow, Glasgow G12 8QQ United Kingdom. Email: [sonya.auer@gmail.com](mailto:sonya.auer@gmail.com)

**1. Material and methods**

**Fish care and feeding regime**

Fish were from wild-origin parents of the River Tweet, Scotland. They were hatched overwinter in the laboratory and fed to satiation while kept in stock tanks in an indoor temperature-controlled (10°C) facility at the University of Glasgow. They were then transferred to individual compartments in a stream tank system located in that same facility [[see 1 for details](#_ENREF_1)] where they were fed commercial trout pellets (5.4 cal/mg, EWOS, West Lothian, UK) on a ration that corresponded to roughly 80% of their sustained maximum intake [[2](#_ENREF_2)]. Specifically, daily caloric intake was computed as a function of body mass (*W*, g) and temperature (*T*, ˚C): 14*W*^0.79^*e* ^0.17^*^T^*. The body mass of each fish was measured every 2-3 weeks while under a mild anaesthetic (benzocaine 40mg/L), and their rations were then adjusted for changes in body size. Once daily, fish were fed and their faecal matter was siphoned from the stream tanks to maintain water quality. Fish were given 8 weeks to acclimate to the food level and stream system before the metabolic and feeding trials began.

**Metabolic rate measurements**

Fish were fasted for 48 hours prior to their standard metabolic rate (SMR) measurements to ensure that the additional metabolic costs of digestion did not inflate estimates of their SMR [[3-5](#_ENREF_3)]. Fish were then placed in individual glass respirometers (400 mL) for 24 hours, with the flow rate set to 2.10 L h^-1^ to allow detection of oxygen consumption rates but not allow oxygen levels to drop below 80% saturation. The oxygen concentration of the water flowing out of the chambers (mg L^-1^) was recorded every 1 min by multichannel oxygen meters and attached sensors (FireStingO2, PyroScience GmbH, Aachen, Germany). An additional fish-free chamber served as a control measure of background respiration rates. Standard metabolic rate (mg O_2_ h^-1^) was calculated for each fish using the equation:

*M*_O2_ = *V*_w_ × (*C*_wO2control_ - *C*_wO2fish_)

where *V*_w_ is the flow rate of water through the respirometry chamber (L h^-1^), and *C*_wO2control_ and *C*_wO2fish_ are the concentrations of oxygen (mg L^-1^) in the outflow of the chambers lacking and containing fish, respectively [[6](#_ENREF_6)]. SMR for each fish was calculated by taking the mean of the lowest 10^th^ percentile of oxygen consumption measurements, and then excluding outliers, i.e. those measurements below 2 standard deviations from this mean [[1](#_ENREF_1)].

The maximum metabolic rate of each fish was then estimated using an exhaustive chase protocol followed immediately by measurement of excess post-exercise oxygen consumption (EPOC) using intermittent flow-through respirometry [[1](#_ENREF_1)]. We chose to elicit MMR using exhaustive chase protocols, rather than other methods such as critical swimming speed, in our study species for several reasons. Similar to other small fishes [[7](#_ENREF_7)], we found that juvenile brown trout are unwilling to swim against a water current in a swim tunnel. However, they are very responsive when chased manually, undergoing many short bursts of very high activity before becoming exhausted. Their willingness or ability to undergo short bursts of activity but not sustained swimming is likely a result of their developmental stage and associated ecology: while older, larger individuals are able to maintain position and drift feed in the open current, juvenile salmonids typically adopt a sit and wait foraging mode, sheltering behind cobbles and only darting out briefly into the current to capture prey [[8](#_ENREF_8), [9](#_ENREF_9)]. As such, exhaustive chase protocols are more likely to elicit MMR and are therefore a more physiologically appropriate and ecologically relevant measurement in our study species.

Briefly, each was fish was chased to exhaustion (< 2 min) against a circular current (600 L h^-1^) in a bucket. Exhaustion was determined when a fish could no longer swim and was unresponsive when picked up by hand. It was then transferred immediately (<10 sec) to a glass respirometry chamber in a closed system were water moved (7.35 L h^-1^) by way of a peristaltic pump through the chamber and then round a circuit of oxygen-impermeable tubing past an oxygen sensing electrode before being returned to the chamber. Each fish was kept in the chamber for 2 minutes, and the decline in water oxygen levels was measured every 2 seconds over that time period using the same type of oxygen meter, sensor, and software as described above. The respirometry chamber was emptied and the system refilled with oxygenated water before measurement of the next fish commenced. Maximum metabolic rate (mg O_2_ h^-1^) was calculated for each fish using the equation:

*M*_O2_ = (*V*_r_ – *V*_f_) × Δ*C*_wO2_/Δt

where *V*_r_ is the volume of the respirometry system (0.120 L), *V*_f_ is the volume of the fish (L) assuming 1 g of fish is equivalent to 1 ml of water, and Δ*C*_wO2_/Δt is the rate at which the oxygen concentration decreased over the 2 minute time period (mg O_2_ L^-1^ h^-1^).

**2. Results**

**Table S1. Parameters (± 1SE) from regression analyses of metabolic rates (mg O_2_ h^-1^) as a function of body mass (*M*, g) in juvenile brown trout (*Salmo trutta*) at 10 ºC.**

|  |  | *M* | df | t | *P* |
| --- | --- | --- | --- | --- | --- |
| Standard metabolic rate | Intercept | 0.33 ± 0.06 | 28 | 5.21 | 0.001 |
|  | Mass | 0.05 ± 0.01 | 28 | 6.15 | < 0.001 |
| Maximum metabolic rate | Intercept | 0.64 ± 0.18 | 28 | 3.64 | 0.001 |
|  | Mass | 0.56 ± 0.03 | 28 | 21.32 | < 0.001 |
| Aerobic scope | Intercept | 0.30 ± 0.20 | 28 | 1.48 | 0.15 |
|  | Mass | 0.52 ± 0.03 | 28 | 17.14 | < 0.001 |

**Figure S1 Standard metabolic rate (SMR), maximum metabolic rate (MMR) and aerobic scope (AS = MMR – SMR), measured as the hourly rate of oxygen consumption as a function of body mass in juvenile brown trout (*Salmo trutta*) at 10 ºC.**

**

**

**Effects of standard and maximum metabolic rate on feeding capacity**

Similar results were obtained when using MMR as a predictor of average meal size instead of AS, with significant positive effects of body mass (*F*_1, 25.1_ = 126.4, *p* <0.001) and mass-independent MMR (*F*_1, 25.0_ = 8.8, *p* = 0.007), but no additional effect of mass-independent SMR (*F*_1, 25.0_ = 0.05, *p* = 0.83).

**3. References**

[1] Auer, S.K., Salin, K., Rudolf, A.M., Anderson, G.J. & Metcalfe, N.B. 2015 The optimal combination of standard metabolic rate and aerobic scope for somatic growth depends on food availability. *Functional Ecology* **29**, 479-486.

[2] Elliott, J. 1976 The energetics of feeding, metabolism and growth of brown trout (Salmo trutta L.) in relation to body weight, water temperature and ration size. *Journal of Animal Ecology* **45**, 923-948.

[3] Secor, S.M. 2009 Specific dynamic action: a review of the postprandial metabolic response. *Journal of Comparative Physiology B* **179**, 1-56.

[4] Higgins, P. & Talbot, C. 1985 Growth and feeding in juvenile Atlantic salmon (Salmo salar L.). In *Nutrition and feeding in fish* (eds. C.B. Cowey, A.M. Mackie & J.G. Bell), pp. 243-263. London, Academic Press.

[5] Rosenfeld, J., Van Leeuwen, T., Richards, J. & Allen, D. 2014 Relationship between growth and standard metabolic rate: measurement artefacts and implications for habitat use and life-history adaptation in salmonids. *Journal of Animal Ecology*, 791-799. (doi:10.1111/1365-2656.12260).

[6] Clark, T.D., Sandblom, E. & Jutfelt, F. 2013 Aerobic scope measurements of fishes in an era of climate change: respirometry, relevance and recommendations. *Journal of Experimental Biology* **216**, 2771-2782.

[7] Killen, S.S., Costa, I., Brown, J.A. & Gamperl, A.K. 2007 Little left in the tank: metabolic scaling in marine teleosts and its implications for aerobic scope. *Proceedings of the Royal Society B: Biological Sciences* **274**, 431-438.

[8] Bachman, R.A. 1984 Foraging behavior of free-ranging wild and hatchery brown trout in a stream. *Transactions of the American Fisheries Society* **113**, 1-32.

[9] Hughes, N.F., Hayes, J.W., Shearer, K.A. & Young, R.G. 2003 Testing a model of drift-feeding using three-dimensional videography of wild brown trout, Salmo trutta, in a New Zealand river. *Canadian Journal of Fisheries and Aquatic Sciences* **60**, 1462-1476. (doi:10.1139/f03-126).

**4. Data used in analyses relating average daily food intake to metabolic rates of juvenile brown trout (*Salmo trutta*).**

| Fish ID | Mass (g) -  Metabolism trial | Standard metabolic rate (mg O_2_ h^-1^) | Maximum metabolic rate (mg O_2_ h^-1^) | Mass (g) -  Feeding trial | Average daily food intake (mg) |
| --- | --- | --- | --- | --- | --- |
| 1 | 6.00 | .62 | 4.23 | 6.848 | 119.46 |
| 2 | 5.00 | .70 | 3.36 | 5.944 | 80.52 |
| 3 | 5.66 | .69 | 3.83 | 6.584 | 69.30 |
| 4 | 4.97 | .68 | 3.14 | 5.631 | 57.42 |
| 5 | 5.35 | .65 | 3.34 | 6.473 | 87.12 |
| 6 | 6.25 | .64 | 4.36 | 6.970 | 77.22 |
| 7 | 7.29 | .66 | 5.20 | 8.480 | 128.04 |
| 8 | 5.61 | .53 | 3.77 | 6.646 | 74.58 |
| 9 | 7.34 | .73 | 4.98 | 8.155 | 114.84 |
| 10 | 4.55 | .57 | 3.14 | 5.172 | 54.78 |
| 11 | 5.38 | .82 | 3.54 | 6.427 | 98.34 |
| 12 | 10.56 | 1.02 | 6.43 | 11.821 | 138.60 |
| 13 | 7.62 | .76 | 4.72 | 8.520 | 99.66 |
| 14 | 6.38 | .65 | 4.65 | 7.350 | 97.68 |
| 15 | 4.47 | .50 | 3.29 | 5.438 | 91.74 |
| 16 | 10.66 | .74 | 7.05 | 11.793 | 186.78 |
| 17 | 5.78 | .65 | 3.79 | 6.584 | 97.02 |
| 18 | 4.07 | .42 | 3.00 | 4.782 | 73.92 |
| 19 | 4.58 | .57 | 2.59 | 5.468 | 85.80 |
| 20 | 5.10 | .63 | 3.50 | 5.936 | 83.16 |
| 21 | 4.66 | .47 | 3.39 | 5.313 | 67.32 |
| 22 | 5.59 | .51 | 3.98 | 6.418 | 90.42 |
| 23 | 7.28 | .64 | 5.16 | 8.286 | 136.62 |
| 24 | 6.97 | .63 | 4.21 | 7.831 | 99.00 |
| 25 | 10.85 | .77 | 6.42 | 12.175 | 157.08 |
| 26 | 5.30 | .45 | 3.59 | 6.146 | 99.66 |
| 27 | 9.09 | .82 | 5.95 | 10.261 | 168.30 |
| 28 | 5.53 | .56 | 3.82 | 6.252 | 111.54 |
| 29 | 9.48 | .90 | 5.53 | 10.525 | 132.00 |
| 30 | 4.28 | .68 | 3.35 | 4.968 | 80.52 |
